# Nutrition in Herbal Plants Used in Saudi Arabia

**DOI:** 10.1155/2020/6825074

**Published:** 2020-04-24

**Authors:** Hanan Almahasheer

**Affiliations:** Department of Biology, College of Science, Imam Abdulrahman Bin Faisal University (IAU), Dammam 31441-1982, Saudi Arabia

## Abstract

Herbs are used for centuries by many people worldwide. This study derives insights into the use and content of herbs that are consumed among Saudi citizens. An online questionnaire was distributed to understand the basic information about Saudi citizens' preference and daily patterns of herbal plants that are usually used as drinks. Moreover, concentrations of fourteen elements in twenty-one herbal plants that were indicated in the previous questionnaire were collected from the local market and then analyzed using an Inductively Coupled Plasma Emission Spectrometry (ICP). Mint leaves were significantly higher in most of the nutrients analyzed, and mint was the most popular drink among participants, followed by green tea and anise. Most of the citizens preferred to drink one cup only at home and believed that herbs are good for their health and potentially could help them to sleep better. The outcomes derived from this research could help future assessments of diet patterns among Saudi citizens.

## 1. Introduction

Herbal plants, hereafter, herbs, are one of the most important sources of many elements that are vital for humans and animals' diet and are used to prevent or treat diseases. These elements are necessary nutrients to their lives and are, therefore, needed in food as the body cannot synthesize them [1]. Herbs play an important role in politics, romance, religion, and health [2], and they are still used by millions of people globally, in particular as medicine as there are more than 50 elements considered as a major component of enzymes and proteins [3]. And optimal uptakes of nutrients may reduce health risk [4]; therefore, their use is increasing globally due to their low side effects when used moderately [5]. Hence, knowing the exact elemental composition of food and other products is important to understand the nutritive value [6], while the use of low nutritive refined food products may affect health [7]. Moreover, some of these are essential nutrients to a certain limit, e.g., iron and copper, whereas others, e.g., lead and cadmium, are poisonous [5].

Most of the herbs are naturally grown in many regions around the planet and used from ancient times [8]. They are also used for culinary [9] cosmetics [10–12], and up to 80% of the world's population depend on plant-derived drugs for medical purposes [13]. Also, green tea is a good example of the use of herbs that started for centuries in China and Japan [6] and is still used nowadays. However, for safety reasons, it is advisable to assess different pollutants before delivering those herbs to the end user (consumer) [14].

Numerous attempts have been made to determine the element's content in plants around the world, e.g., Nigeria [15] and Malaysia [16]. However, a quick search in Scopus with the keywords “herbal plant^*∗*^” and “nutrient^*∗*^” revealed that most of the published papers are from Asia, i.e., India, China, Iran, and Malaysia. Saudi Arabia's market is rich with various herbal plants that citizens are using as “traditional medicine,” many of those are coming from Asia and Africa, while the rest are harvested locally. Studies concerning elemental content of these herbs that are acquired from the Saudi market are rare, whereas herbal preference among citizens and their knowledge base have not hitherto been performed. As a result, the outcomes of this research would serve as a base for further assessments related to herbal plants in Saudi Arabia and describe patterns of practices among Saudi citizens.

## 2. Materials and Methods

### 2.1. Questionnaire

To assess the public preference regarding herbal plants that are usually used on a daily basis by people in Saudi Arabia, a survey was carried out online in 2018. The participants were asked before starting the survey if they do drink herbal tea; if no, the survey was terminated. The panel respondents who were above 18 years old were invited to participate via Google survey online (https://gsuite.google.com/products/forms/). However, only 44 participants were under 18 which corresponds to 4% only; therefore, their answers were not excluded from the results. Demographic characteristics (i.e., questions about sex, age, and educational stage were asked to identify the range of participants) are shown in Supplementary Table S1. The questionnaire was designed to include the following: From where do you get your herbs, why do you drink herbal tea, does it help you sleep, where do you drink it, how many cups per day, how many scoops of the herb do you use per cup, and which herbs do you drink, along with options to choose from which are in Supplementary Table S2. The questionnaire was in the Arabic language; then, the results were translated to English.

### 2.2. Collection of the Samples

**Figure 1:**
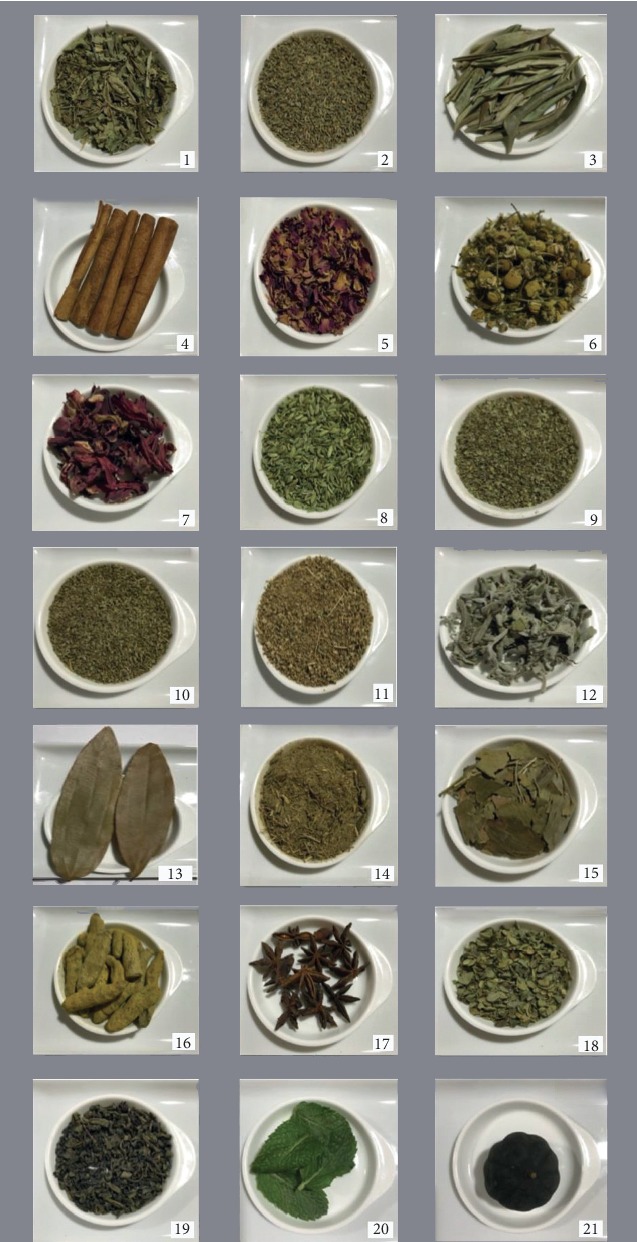
Images of herbal plants acquired and analyzed, which correspond to plants in Table 1.

Twenty-one herbal plants that were indicated in the previous questionnaire (Table 1 and Figure 1) were collected from a local market in Dammam, Saudi Arabia, in 2018. The material was already dried; therefore, three replicated samples (*n* = 3) from each herb were directly ground in an agate mortar. The scientific name and family of each herb were further investigated using Encyclopedia Britannica (http://www.britannica.com) and Herbal Encyclopedia (https://www.cloverleaffarmherbs.com).

### 2.3. Chemical Analysis and Data Quality

Plant samples were already dried, then ground using a granite mortar, and photographed; then, 0.5 grams of the plant sample (i.e., leaves, stem, or seeds) was digested with 5 mL of concentrated HNO_3_ and 2 mL of H_2_O_2_ in polyethylene tubes at digestion systems for 2 hours at 100°C [17]. The digested samples were left to cool, then diluted to 45 mL with Milli-Q water (18.2 Ω/cm), then filtered with a Whatman filter paper of 44-micron size. Concentrations of fourteen elements: calcium (Ca), chromium (Cr), copper (Cu), iron (Fe), iodine (I), potassium (K), magnesium (Mg), manganese (Mn), molybdenum (Mo), sodium (Na), nickel (Ni), phosphorus (P), selenium (Se), and zinc (Zn), were analyzed using Inductively Coupled Plasma Emission Spectrometry (Shimadzu, model 9820). To confirm the quality of the analysis, replicates and PanReac AppliChem multielement standard solution between the 20 samples were used. The analytical recovery of the standards and the duplicated samples is reported in Table 1.

### 2.4. Statistical Analysis

Descriptive statistics and general linear models were used to test the effects of differences among herbs for every single element as well as Tukey's HSD (honestly significant difference) post hoc test was used to assess pairwise differences. Moreover, assuming that the average consumption of herbs is one spoon equivalent of 0.5 g, the estimated dietary intake (mg day^−1^) based on 70 kg body weight was calculated by dividing the nutrient concentration (mg kg^−1^) by 70. All statistics were computed using JMP v12 (Table 2).

## 3. Results

The range of participants who answered the online survey that was distributed through the social media was from 12 to 70 years old, with about 1200 participants in about 10 days (only 44 participants were under 18 years old which corresponds to 4% only; therefore, their answers were not excluded from the results). Female participants were the highest comprising about 77%, while male participants were only 23%. Details of age and sex along with the education level of participants are summarized in Supplementary Table S1.

**Figure 2:**
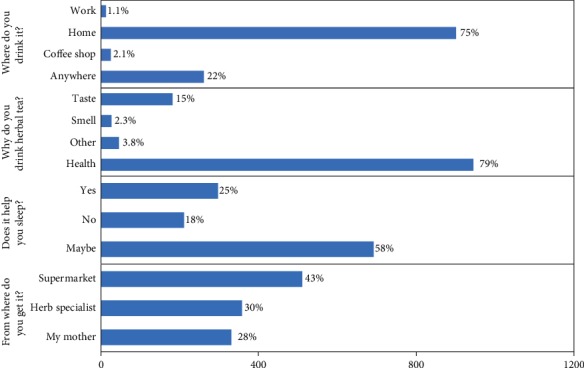
Analyses of survey questions (from where do you get your herbs, why do you drink herbal tea, does it help you sleep, and where do you drink it?).

The majority of participants, i.e., 75% of the participants, have their herbal tea at home, and 22% of the participants drink it anywhere, whereas only 2% and 1% have it at the coffee shop or at work. Moreover, 79% of the participants believe that drinking herbal tea is good for health, while 15% prefer it just for the taste, 2% chose it for the smell, and 4% have other reasons. Interestingly, about 83% answered yes or maybe to the question about if they believe if the herbal tea would help them to sleep, while only 18% answered with no. Additionally, about 58% answered that they get it from their mothers or herb specialists, whereas 43% from the supermarket (Figure 2 and Supplementary Table S2).

**Figure 3:**
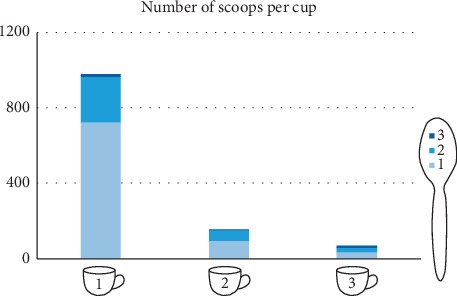
Analyses of survey questions (how many cups do you consume per day, and how many scoops of herb do you use per cup?).

Furthermore, 71% (i.e., 852 participants) of the 1200 preferred to drink only one cup of herb a day; 60% out of those (852 participants) prepared it with only one spoon, 8% prepared it using two spoons, and only 3% used three spoons. Moreover, 20% (i.e., 240 participants) of 1200 preferred to drink two cups of herb a day; 20% of those (240 participants) prepared it with only one spoon, 5% prepared it using two spoons, and only 2% used three spoons. Finally, only 28 participants preferred three cups a day, and 1%, 0.3%, and 0.8% preferred one, two, and three spoons per cup, respectively (Figure 3 and Supplementary Table S2).

**Figure 4:**
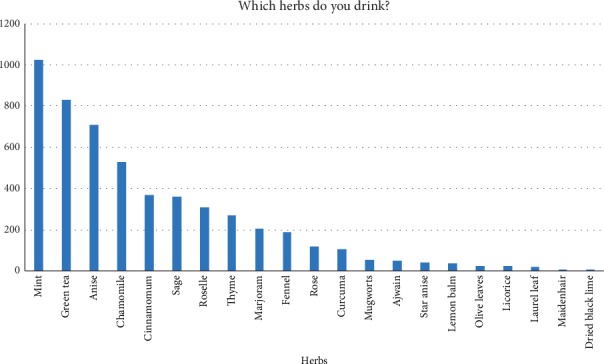
Analyses of the survey question, (which herbs do you drink?).

The majority of the Saudi participants (about 85%) preferred to drink mint, followed by 69% favoring green tea and then 59% with anise. Moreover, Chamomile, Cinnamomum, Sage, Roselle, Thyme, Marjoram, and Fennel were intermediate with 44, 31, 30, 26, 22, 17, and 16% respectively while the rest (i.e., rose, curcuma, mugworts, ajwain, star anise, lemon balm, olive leaves, licorice, laurel leaf, maidenhair, and dried black lime) were ≤10% (Figure 4).

Most of the elements analyzed, i.e., Ca, Cu, Fe, Cr, Mn, Ni, and Se, were significantly higher in mint, maidenhair, green tea, dried black lime, and laurel leaf whereas Zn, Na, and Mg were significantly higher in mugworts, and K was significantly higher in both mugworts and curcuma. Moreover, P, Mo, and I were significantly higher in lemon balm, dried black lime, and mint, respectively (Table 3, Tukey's HSD, *P* < 0.05).

## 4. Discussion

The Recommended Dietary Allowance (RDA) is the sufficient amount of required nutrients that are needed for healthy individuals, which are established by the Food and Nutrition Board [18]. Herbal plants are one of the major resources of these nutrients and, therefore, the amount of nutrients in plants is one of the criteria that makes it favorable to use [6], although the paucity of these nutrients can lead to disease [1]. Many of them are essential nutrients for humans; e.g., iron is basic to hemoglobin and many enzymes, while calcium and phosphorous are essential components to bones, magnesium is important to all biosynthetic processes, and zinc is basic to many enzymes that are involved in metabolic pathways [19].

The estimated dietary intake (mg day^−1^) in this study compared to the RDA varied among different elements. That is, Ca, K, Mg, Na, P, and Zn were within the recommended range of intake for males and females (Table 4) while Cr, Cu, I, Mo, and Se were higher than the range of the reported values and finally Fe and Mn were intermediate with seven herbs (i.e., black lime, green tea, laurel leaf, maidenhair, mint, mugworts, and thyme; Table 4) out of twenty-one being higher than the RDA values reported by Dell Valle et al. [20].

Moreover, in this study, two-thirds of the participants were females, indicating a higher rate of use among females; this rate of female users was observed by the findings of Alghamdi et al. [21]. The growing interest of females to herbal drinks, e.g., tea, might be due to the daily news of their benefits that ranged from the protection of hip structure in elderly women [22] to cancer potential treatments and risks caused by diabetes [23].

While there is no explanation why the majority drink only one cup of herbal tea preferring it at home compared to the work place, and around half of the participants acquire it from their moms or specialists, yet, in general, about 79% of the participants believed that herbal tea is good for their health compared to smell and taste, and 83% believed that it may help them sleep. Similarly, a recent study on two hundred patients in Saudi Arabia found that 76% of the patients with chronic illness had used herbal medicine [21], compared to a lower range of 10 to 52% in western countries [24]. And a higher range of migrants of about 75% uses herbal medicine in the west [25].

Mint was the most popular herb among Saudi citizens, followed by green tea and anise. In Australia, mint comes as number seven of most sold herbs and spices in the supermarket for 2013 with about $2151500 of sales value [26]. However, many studies documented the effect of green tea as a potential treatment preventing cancer [27, 28]. Furthermore, most of the nutrients, i.e., Ca, Cu, Fe, Cr, Mn, Ni, and Se, were significantly higher in mint, maidenhair, green tea, dried black lime, and laurel leaf, whereas nutrients' concentrations in this study, particularly, Zn, Na, K, and Mg, were significantly higher in mugworts compared to other herbs, which is coherent with early Chinese beliefs of the medical benefits of mugworts, as well as France in the middle ages, where they used it to protect babies from cold [2].

Nevertheless, these herbs must be used with care, as many reports have documented the presence of toxic heavy metals and undeclared pollutants, e.g., in Asia [29–31] and Africa [32, 33].

## 5. Conclusion

The concentration of nutrients carried out was depending on the plant species analyzed; most of the herbs analyzed were within the RDA limits. However, participants who selected many cups per day or many spoons per cup could be at risk of bioaccumulation. In this study, mint leaves were significantly higher in most of the nutrients analyzed, and mint was the most popular drink among Saudi citizens, followed by green tea and anise.

## Figures and Tables

**Table 1:** Herbal plants used in this study.

Number	Name		Family	Part assayed
	Common	Scientific		
1	Lemon balm	*Melissa officinalis*	Labiatae	Leaves
2	Anise	*Pimpinella anisum*	Umbelliferae	Seeds
3	Olive leaves	*Olea europaea*	Oleaceae	Leaves
4	Cinnamomum	*Cinnamomum verum*	Lauraceae	Tree bark
5	Rose	*Rosa species*	Rosaceae	Flowers
6	Chamomile	*Matricaria chamomilla*	Asteraceae	Flowers
7	Roselle	*Hibiscus sabdariffa*	Malvaceae	Flowers
8	Fennel	*Foeniculum vulgare*	Umbelliferae	Seeds
9	Marjoram	*Origanum majorana*	Labiatae	Leaves
10	Ajwain	*Trachyspermum ammi*	Apiaceae	Seeds
11	Mugworts	*Artemisia vulgaris*	Asteraceae	Leaves and twigs
12	Sage	*Salvia officinalis*	Labiatae	Leaves and twigs
13	Laurel leaf	*Laurus nobilis*	Lauraceae	Leaves
14	Licorice	*Glycyrrhiza glabra*	Fabaceae	Roots
15	Maidenhair	*Adiantum*	Polypodiaceae	Leaves and twigs
16	Curcuma	*Curcuma longa*	Zingiberaceae	Rhizome
17	Star anise	*Illicium verum*	Schisandraceae	Seeds
18	Thyme	*Thymus vulgaris*	Lamiaceae	Leaves
19	Green tea	*Camellia sinensis*	Theaceae	Leaves
20	Mint	*Mentha*	Labiatae	Leaves
21	Dried black lime	*Citrus aurantifolia*	Rutaceae	Fruit

**Table 2:** Summary of certified standards and the duplicated samples measured, along with the selected wavelength of each element analyzed.

Element	Symbol	Wavelength	% Recovery	
			Standard	Duplicated samples
Calcium	Ca	220.86	99	86
Chromium	Cr	205.55	100	113
Copper	Cu	261.84	98	98
Iron	Fe	259.94	101	82
Iodine	I	178.28	112	89
Potassium	K	766.49	86	93
Magnesium	Mg	279.55	98	120
Manganese	Mn	260.57	96	88
Molybdenum	Mo	202.03	102	100
Sodium	Na	589.59	87	94
Nickel	Ni	231.60	100	94
Phosphorus	P	213.62	96	86
Selenium	Se	203.99	95	89
Zinc	Zn	213.86	98	95

**Table 3:** Average ± standard error (SE) of fourteen elemental concentrations (mg kg^−1^) for twenty-one herbs (*n* = 3) collected from Saudi Arabia.

	Ca	Cr	Cu	Fe	I	K	Mg	Mn	Mo	Na	Ni	P	Se	Zn
Sage	429 ± 5^de^	339 ± 5^bc^	376 ± 3^e^	430 ± 4^d^	1210 ± 17^defg^	1970 ± 81^ef^	1467 ± 20^cd^	107 ± 2^c^	559 ± 8^de^	998 ± 80^defg^	388 ± 5^c^	1127 ± 23^abcde^	981 ± 12^bcd^	247 ± 5^bc^
Ajwain	345 ± 57^de^	203 ± 120^c^	269 ± 125^e^	159 ± 86^d^	724 ± 169^ghij^	1152 ± 179^f^	556 ± 398^de^	125 ± 9^c^	424 ± 62^de^	333 ± 173^fg^	233 ± 109^c^	840 ± 405 ^def^	569 ± 116^efgh^	185 ± 15 ^ef^
Anise	497 ± 6^cde^	299 ± 5^bc^	660 ± 14^de^	355 ± 5^d^	1087 ± 17^efgh^	9147 ± 116^b^	1503 ± 19^cd^	156 ± 6^c^	463 ± 8^de^	1303 ± 39^defg^	332 ± 5^c^	1447 ± 18^abc^	730 ± 8^def^	194 ± 3^ef^
Chamomile	323 ± 3^e^	274 ± 3^c^	308 ± 9^e^	360 ± 10^d^	1165 ± 49^defgh^	3855 ± 193^de^	981 ± 225^cde^	119 ± 3^c^	416 ± 1^de^	746 ± 86^efg^	309 ± 3^c^	1067 ± 169^bcde^	580 ± 18^efg^	190 ± 10 ^ef^
Cinnamomum	309 ± 27^e^	156 ± 49^c^	153 ± 24^e^	69 ± 24^d^	608 ± 34^ghij^	2213 ± 179^ef^	249 ± 12^e^	138 ± 16^c^	302 ± 38^e^	390 ± 80^fg^	169 ± 21^c^	433 ± 34^f^	417 ± 32^fghi^	120 ± 7^g^
Curcuma	280 ± 25^e^	224 ± 4^c^	178 ± 41^e^	235 ± 16^d^	550 ± 53 ^hij^	12133 ± 120^a^	907 ± 346^de^	135 ± 8^c^	327 ± 8^e^	342 ± 85^fg^	227 ± 8^c^	592 ± 14^ef^	388 ± 21^ghi^	121 ± 2^g^
Dried black lime	2693 ± 97^a^	1327 ± 48^a^	2977 ± 80^ab^	2533 ± 77^a^	2280 ± 76^ab^	6737 ± 155^bc^	1014 ± 36^cde^	834 ± 30^a^	1563 ± 304^a^	7467 ± 149^bc^	3050 ± 235^a^	1257 ± 43^abcd^	1317 ± 38^a^	251 ± 9^abc^
Fennel	610 ± 9^cde^	321 ± 7^bc^	593 ± 55^de^	293 ± 11^d^	1160 ± 20^defgh^	8750 ± 163^b^	1980 ± 21^bc^	177 ± 6^c^	516 ± 11^de^	2488 ± 998^def^	340 ± 8^c^	1587 ± 28^ab^	916 ± 23^cd^	197 ± 4^def^
Green tea	2623 ± 90^ab^	1293 ± 43^a^	3150 ± 121^a^	2607 ± 98^a^	2243 ± 97^ab^	7073 ± 286^bc^	990 ± 34^cde^	813 ± 28^a^	1267 ± 41^ab^	8433 ± 398^bc^	2860 ± 115^a^	1230 ± 55^abcd^	1297 ± 38^ab^	248 ± 10^bc^
Laurel leaf	2697 ± 85^a^	1327 ± 41^a^	2853 ± 186^ab^	2480 ± 121^ab^	2220 ± 145^abc^	6193 ± 315^cd^	1011 ± 31^cde^	834 ± 26^a^	1157 ± 66^abc^	6273 ± 496^c^	2607 ± 172^a^	1220 ± 58^abcd^	1287 ± 35^ab^	244 ± 9^bcd^
Lemon balm	453 ± 93^cde^	429 ± 2^bc^	388 ± 96^e^	555 ± 1^d^	1593 ± 3^cde^	1227 ± 116^f^	872 ± 602^de^	140 ± 1^c^	726 ± 3 ^cde^	155 ± 62^fg^	146 ± 93^c^	1673 ± 13^a^	1200 ± 6^abc^	289 ± 3^ab^
Licorice	1610 ± 538^abc^	416 ± 16^bc^	823 ± 44^de^	573 ± 51^d^	1027 ± 27^efghi^	3847 ± 102^de^	2560 ± 92^b^	196 ± 4^c^	608 ± 19^de^	3067 ± 397^de^	472 ± 23^c^	1110 ± 32 ^abcde^	944 ± 27^cd^	225 ± 9 ^cde^
Maidenhair	2733 ± 67^a^	1347 ± 33^a^	3310 ± 80^a^	2723 ± 67^a^	2337 ± 85^ab^	7557 ± 169^bc^	1030 ± 25^cde^	845 ± 20^a^	1317 ± 33^ab^	9243 ± 167^b^	3047 ± 69^a^	1287 ± 30^abcd^	1340 ± 32^a^	257 ± 5^abc^
Marjoram	301 ± 109^e^	343 ± 27^bc^	425 ± 43^e^	451 ± 65^d^	1192 ± 150^defg^	2187 ± 175^ef^	1280 ± 52^cde^	123 ± 6^c^	520 ± 46^de^	858 ± 144^efg^	386 ± 53^c^	1130 ± 166^abcde^	874 ± 93 ^de^	232 ± 9 ^cde^
Mint	2753 ± 37^a^	1357 ± 19^a^	3327 ± 34^a^	2720 ± 25^a^	2383 ± 46^a^	7727 ± 91^bc^	1040 ± 15^cde^	852 ± 11^a^	1313 ± 18^ab^	9560 ± 140^a^	3087 ± 39^a^	1297 ± 18^abcd^	1347 ± 24^a^	259 ± 3^abc^
Mugworts	1213 ± 18^cde^	560 ± 7^bc^	1560 ± 40^cd^	779 ± 14^cd^	1497 ± 19^def^	12667 ± 133^a^	6083 ± 87^a^	335 ± 7^bc^	899 ± 10^bcd^	15267 ± 145^a^	655 ± 8^bc^	1427 ± 19^abcd^	1320 ± 15^a^	300 ± 2^a^
Olive leaves	568 ± 79^cde^	225 ± 42^c^	403 ± 71^e^	177 ± 33^d^	883 ± 75^fghi^	2467 ± 413^ef^	971 ± 130^cde^	128 ± 13^c^	437 ± 38^de^	407 ± 102^fg^	378 ± 87^c^	888 ± 124^cdef^	824 ± 125^de^	171 ± 12^f^
Rose	143 ± 1^e^	143 ± 1^c^	41 ± 5^e^	77 ± 4^d^	242 ± 22^j^	587 ± 19^f^	564 ± 121^de^	68 ± 2^c^	367 ± 167^e^	576 ± 41^fg^	126 ± 0^c^	418 ± 9^f^	167 ± 11^i^	86 ± 1^g^
Roselle	285 ± 70^e^	349 ± 5^bc^	419 ± 17^e^	375 ± 34^d^	1210 ± 15^defg^	1980 ± 26^ef^	1490 ± 26^cd^	243 ± 5^c^	576 ± 9^de^	659 ± 71^fg^	440 ± 17^c^	1073 ± 12 ^abcde^	977 ± 6 ^bcd^	244 ± 3 ^bcd^
Star anise	204 ± 7^e^	153 ± 5^c^	191 ± 17^e^	139 ± 56^d^	395 ± 27^ij^	1122 ± 123^f^	514 ± 42^de^	172 ± 3^c^	352 ± 117^e^	54 ± 26^g^	162 ± 5^c^	427 ± 12^f^	254 ± 7^hi^	97 ± 3^g^
Thyme	150 ± 789^bcd^	762 ± 378^b^	1949 ± 876^bc^	1587 ± 735^bc^	1745 ± 427^bcd^	4185 ± 1891^de^	871 ± 184^de^	604 ± 243^ab^	902 ± 190^bcd^	3298 ± 1523^d^	1600 ± 761^b^	1055 ± 123^bcde^	1045 ± 171^abcd^	215 ± 26^cdef^
*F* ratio *P* value	22^*∗∗*^	26^*∗∗*^	35^*∗∗*^	36^*∗∗*^	31^*∗∗*^	67^*∗∗*^	38^*∗∗*^	31^*∗∗*^	17^*∗∗*^	93^*∗∗*^	38^*∗∗*^	10^*∗∗*^	39^*∗∗*^	43^*∗∗*^

The results (*F* ratio and *P*value: ^*∗*^=0.05 > *P* > 0.01; ^*∗∗*^=*P* < 0.01) from ANOVA. Different letters a, b, c, and d indicate significant differences among different herbs for every single element (Tukey's HSD multiple comparison post hoc test, *P* < 0.05).

**Table 4:** Median nutrient intake values by herbs based on 70 kg body weight (mg day^−1^).

	Ca	Cr	Cu	Fe	I	K	Mg	Mn	Mo	Na	Ni	P	Se	Zn
Sage	6	5	5	6	17	27	21	2	8	14	6	16	14	4
Ajwain	4	2	5	2	8	15	3	2	5	5	4	6	7	3
Anise	7	4	10	5	15	132	21	2	7	18	5	21	10	3
Chamomile	5	4	4	5	17	55	14	2	6	11	4	15	8	3
Cinnamomum	5	2	2	1	9	32	4	2	5	6	2	7	6	2
Curcuma	4	3	2	4	8	174	10	2	5	5	3	9	5	2
Dried Black lime	40	20	43	37	33	97	15	12	18	105	40	19	19	4
Fennel	9	5	9	4	17	124	28	3	8	49	5	23	13	3
Green tea	37	18	45	37	33	101	14	12	18	120	41	17	19	4
Laurel leaf	39	19	42	35	31	91	15	12	17	91	38	17	18	4
Lemon Balm	8	6	5	8	23	17	5	2	10	2	1	24	17	4
Licorice	22	6	12	9	14	54	37	3	9	39	7	16	13	3
Maidenhair	40	20	48	40	34	110	15	12	19	133	44	19	19	4
Marjoram	4	5	6	6	16	30	18	2	7	11	5	17	12	3
Mint	40	20	48	39	34	111	15	12	19	137	44	18	19	4
Mugworts	17	8	23	11	22	183	88	5	13	219	9	21	19	4
Olive leaves	8	3	7	3	13	41	14	2	6	5	5	13	13	3
Rose	2	2	1	1	3	8	9	1	3	8	2	6	2	1
Roselle	5	5	6	6	17	28	21	3	8	9	6	15	14	4
Star anise	3	2	3	3	5	18	7	2	3	1	2	6	4	1
Thyme	22	12	40	30	30	85	15	12	15	63	30	16	16	3
Males (9 to >70 y)^*∗*^	1000–1300	0.03–0.04	0.07–0.09	8–11	0.1–0.2	4500–4700	240–420	1.9–2.3	0.03–0.05	1200–1500	—	700–1250	0.1	8–11
Females (9 to >70 y)^*∗*^	1000–1300	0.02–0.03	0.07–0.09	8–18	0.1–0.2	4500–4700	240–320	1.6–1.8	0.03–0.05	1200–1500	—	700–1250	0.1	8–9

^*∗*^RDA values from [20]

## Data Availability

The data used to support the findings of this study are included within the supplementary information files.

## Supplementary Materials

Supplementary Materials Supplementary Tables S1 and S2: questions about sex, age, and educational stage were asked to identify the range of participants.
